# sc-MULTI-omics approach in nano-rare diseases: understanding the pathophysiological mechanism of Mulvihill-Smith Syndrome

**DOI:** 10.1007/s10142-025-01608-y

**Published:** 2025-05-09

**Authors:** Angelika Riess, Cristiana Roggia, Antje Schulze Selting, Vladislav Lysenkov, Stephan Ossowski, Nicolas Casadei, Olaf Riess, Yogesh Singh

**Affiliations:** 1 Institute of Medical Genetics & Applied Genomics, University Hospital Tübingen, Tübingen University, Tübingen, Germany; 2 NGS Competence Centre Tübingen, University Hospital Tübingen, Tübingen University, Tübingen, Germany; 3 Research Institute of Women’s Hospital, University Hospital Tübingen, Tübingen University, Tübingen, Germany

**Keywords:** Mulvihill-Smith Syndrome, sc-MULTI-omics, PBMCs, Nano-rare disease

## Abstract

Mulvihill-Smith Syndrome (MSS) is a clinically complex and genetically unsolved nano-rare disorder with only 12 patients reported in the literature. Most patients (91%) have immunological impairments, succumb to infection, and might develop cancer later in life. Its pathogenesis remains elusive and therapeutic options are limited. We used single-cell MULTI-omics (sc-MULTI-omics), combining transcriptomics (gene expression, TCR, and BCR repertoire) and proteogenomic (Cellular Indexing of Transcriptomes and Epitopes by Sequencing; CITE-seq), to decipher the pathophysiology of nano-rare disease patient. We report a new patient who is a 16-year-old girl. She had an increased leukocyte counts and typical manifestations of MSS such as short stature, older appearance, multiple pigmented nevi, microcephaly, monolateral keratoconus, Marcus-Gunn syndrome, hearing loss, vitamin D deficiency, mild hypercortisolism, and diabetes mellitus with very high insulin resistance (T3DM). sc-MULTI-omics CITE-seq showed that the MSS patient had increased central memory CD4^+^ T cells as well as effector memory CD8^+^ T cells, whilst reduced naïve T cells (both CD4^+^ and CD8^+^ T cells). Furthermore, we identified genes and pathways associated with the progeria-like phenotype, inflammation, and cancer progression, which may contribute to the clinical signs of MSS. sc-MUTLI-omics CITE-seq analyses improve our understanding of complex human disease pathophysiology and provides an alternative approach in personalized medicine in nano-rare disease.

**Graphical abstract:**

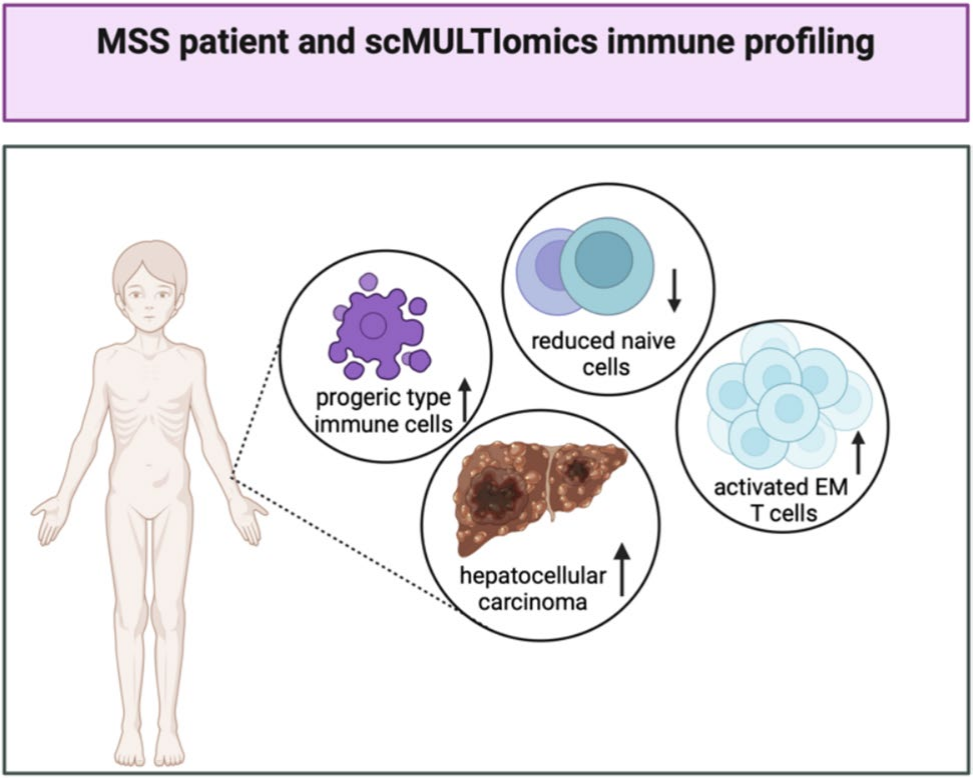

**Supplementary Information:**

The online version contains supplementary material available at 10.1007/s10142-025-01608-y.

## Background

Mulvihill-Smith Syndrome (MSS) belongs to progeroid disorders and has been described only in 12 patients (NORD 2024; Tavdy et al. 2024; Passarelli, et al. 2018). The suspected mode of inheritance is autosomal recessive, and the disorder is mostly sporadic in nature (NORD 2024; Tavdy et al. 2024; Passarelli, et al. 2018). This nano-rare disorder is characterised by intrauterine growth retardation, short stature, microcephaly, premature aging, bird-like facies with a lack of facial subcutaneous fat, multiple pigmented nevi, sensorineural hearing loss, orofacial or dental abnormalities, ophthalmic problems, sleep disorders, diabetes mellitus, and variable intellectual disability (Passarelli, et al. 2018; Crooke 2022; Bartsch et al. 1994). In addition, four patients were diagnosed in early age (between 16–28 years) with solid tumors (gastric, tongue, pancreas, and melanoma) (NORD 2024; Bartsch et al. 1999). A genetic cause of the disease is strongly suspected; however, to date, no causative gene has been identified.

Most of the reported patients had immunological impairment (NORD 2024; Passarelli, et al. 2018; Ohashi et al. 1993). It was demonstrated that the ability of the body’s immune system to fight invading organisms appears to be compromised in MSS patients (Bartsch et al. 1994, 1999; Ohashi et al. 1993). Blood analyses revealed reduced levels of key cells of the immune system, especially of T and B cells (Ohashi et al. 1993). An impaired immune system is perceived as a hallmark characteristic of MSS and is often used to distinguish it from other premature aging diseases, such as Cockayne syndrome (NORD 2024; Ohashi et al. 1993; Zayoud 2024). In addition, recurrent infections have been reported in most but not all MSS patients (Bartsch et al. 1994; Ohashi et al. 1993). We identified another MSS patient (16 years age, girl) with characteristic clinical features and a higher lymphocyte count. Before the patient developed liver cancer, blood was collected for single cell MULTI-omics Cellular Indexing of Transcriptomes and Epitopes by Sequencing (sc-MULTIomics-CITE-seq; transcriptomics and proteogenomic) to characterize the immune response.

## Materials and methods

### Patient demographics

Both, MSS patient and mother, signed the consent for the Genome + study (ClinicalTrial.gov-Nr.: NCT04315727 and project Nr.: 635/2014BO1). MSS was diagnosed based on clinical findings of short stature, older appearance, multiple pigmented nevi, and microcephaly at the age of 1 year (2006) and she is currently 19 years old. In this duo study, the patient with MSS and her mother were recruited for sc-MULTIomics-RNA-seq analysis on 06/2022. A few months later, the MSS patient was diagnosed with T3DM (10/2022), and 6 months afterward (12/2022) with HCC.

### Blood collection, PBMC isolation and sample preparation for single cell cellular indexing of transcriptomics and epitopes by sequencing (CITE-seq)

PBMCs were isolated using the standard Ficoll method (Singh et al. 2021). We used fresh PBMCs (0.5 × 10^6^) for the single cell CITE-seq preparation. PBMCs were incubated with a mixture of 137 feature barcode (FB) antibodies labelled with unique molecular barcodes, including seven isotype barcode antibodies (TotalSeq™-C Human Universal Cocktail, V1.0; #399905, BioLegend, USA) as recommended by the manufacturer (detailed information is available in supplementary materials and method section). Further, single cell [CITE-seq, T cell receptor (TCR) and B cell receptors (BCR)] (sc-MULTIomics-seq) library preparation and sequencing was performed as recommended by the 10 × Genomics’ protocol using 5’GEM v2 kits PN-1000425 (gene expression (GEX), feature barcode antibodies (FB), TCR, and BCR) with slight modifications. Sequencing was performed using a paired-end 200 bp reading strategy on an Illumina platform. An appropriate amount was loaded onto the sequencing S2 cell flow (Illumina) to obtain > 35,000 reads/cell for GEX, > 10,000 reads/cell for FB, > 10,000 reads/cell for TCR, and > 10,000 reads/cell for BCR.

### Data analysis for sc-MULTIomics-RNA-seq experiment

Sequencing output files (FASTQ files) were processed using a 10 × Genomics's cell ranger pipeline to obtain count matrix files (filtered feature barcode matrices) for the downstream data analysis. Filtered feature gene expression matrices generated per sample were analyzed with the Seurat 5.01 package in RStudio (4.4.2). The filtering steps for high-quality single cells included cells expressing > 200 genes, cells with > 800 detected molecules (the total number of unique molecular indices (UMIs) detected), and cells with a mitochondrial gene count percentage of < 10%. Based on the clean scRNA-seq data after quality control (QC) checks, gene expression profiles were normalized for each cell using the Log-Normalization method with the Seurat NormalizeData function. The FindVariableFeatures function was applied to identify highly variable genes using the default parameters. Next, we scaled the data using the ScaleData function and performed principal component analysis (PCA) on the scaled data using the RunPCA function with default parameters. A canonical correlation analysis (CCA) integration algorithm was used for batch correction (Hao, et al. 2021; Stuart, et al. 2019). We constructed a shared nearest neighbour graph using the FindNeighbors function and clustered cells using the Louvain algorithm with the FindClusters function, with the resolution set to 0.8, based on CCA integration reduction (Arevalo et al. 2024). Finally, the RunUMAP function facilitates the visualization of all cells. The FindAllMarkers function was used to identify the marker genes for each cluster. Clusters were then classified and annotated based on the marker gene expression of each cell subset. In the process of mapping query datasets to annotated references in Seurat, we mapped our PBMC dataset obtained from the MSS patient and control to the CITE-seq (proteogenomic) reference of 162,000 PBMC measured using 228 antibodies (Hao, et al. 2021). We loaded the published reference data set (Hao, et al. 2021) and visualized the pre-computed UMAP.

### Identification of differential expressed genes across the MSS patient and control

To perform comparative analyses across the MSS patient and control group, we used the bulk of Seurat’s differential expression features which was accessed through the FindMarkers function. By default, Seurat performs differential expression (DE) testing based on the non-parametric Wilcoxon rank-sum test. To test for DE genes between the two specific groups of cells (MSS patient and control), we specified the ident.1 and ident.2 parameters. Differentially expressed genes (DEGs) from each cell subtype were filtered based on the following criteria: absolute fold change ≥ 0.2 and adjusted *p* value ≤ 0.05; based on false discovery rate (FDR) correction.

### Metascape data analysis

The Metascape web browser tool utilizes a well-adopted hypergeometric test and Benjamini–Hochberg *p*-value correction algorithm to identify all ontology terms which encompass a statistically greater number of genes in common with an input list than expected by chance (Zhou et al. 2019). Thus, we used the input of highly upregulated genes (top 100 genes from the DEGs list) from monocytes I, CD8^+^ EM, and CD4^+^ EM T cells that were derived from the MSS patients compared with control based on log_2_FC and adjusted *p* value correction utilizing the Bonferroni correction method using all features in the dataset. These genes were subjected to Metascape web browser analysis (Zhou et al. 2019) to identification of different biological pathways.

### Gene set enrichment analysis (GSEA) for Gene Ontology (GO) and Kyoto Encylopedia of Genes and Genomes (KEGG) pathways

GSEA was used to identify whether a pre-defined set of genes (log_2_FC ≥ 0.2 and adjusted *p* value ≤ 0.05) in a particular subset of cells showed statistically significant, concordant differences between the MSS patient and control. Significantly activated or inhibited KEGG pathways were selected with absolute NES ≥ 1 and adjusted *p* value ≤ 0.05, as the threshold. For each cell subset, Gene ontology (GO) enrichment analysis was performed on the previously selected DEGs. The significantly changed GO entries were filtered with adjusted *p* value ≤ 0.05 and count ≥ 2 as threshold.

### TCR and BCR repertoire sequencing and analyses

Most of the downstream analysis for TCR and BCR repertoire was performed using scRepetoire pipeline (Borcherding et al. 2020) and described earlier by others (Andreatta et al. 2023).

### Statistical analyses

Statistical analyses were performed using R software (v.4.3.0). This is a single patient-specific report duo study report. Therefore, major statistical analysis was not performed. However, significant differences in gene expression were determined by non-parametric Wilcoxon rank sum test and the *p* values were adjusted with FDR utilizing the Bonferroni correction method.

### Data availability

All the detailed analysis pipeline is available on github repositories (https://github.com/ysinghbt/MSS) and data is available on Zenodo (DOI: 10.5281/zenodo.10894666). Further information on data availability can be provided upon reasonable request from the corresponding authors.

## Results

**Fig. 1:**
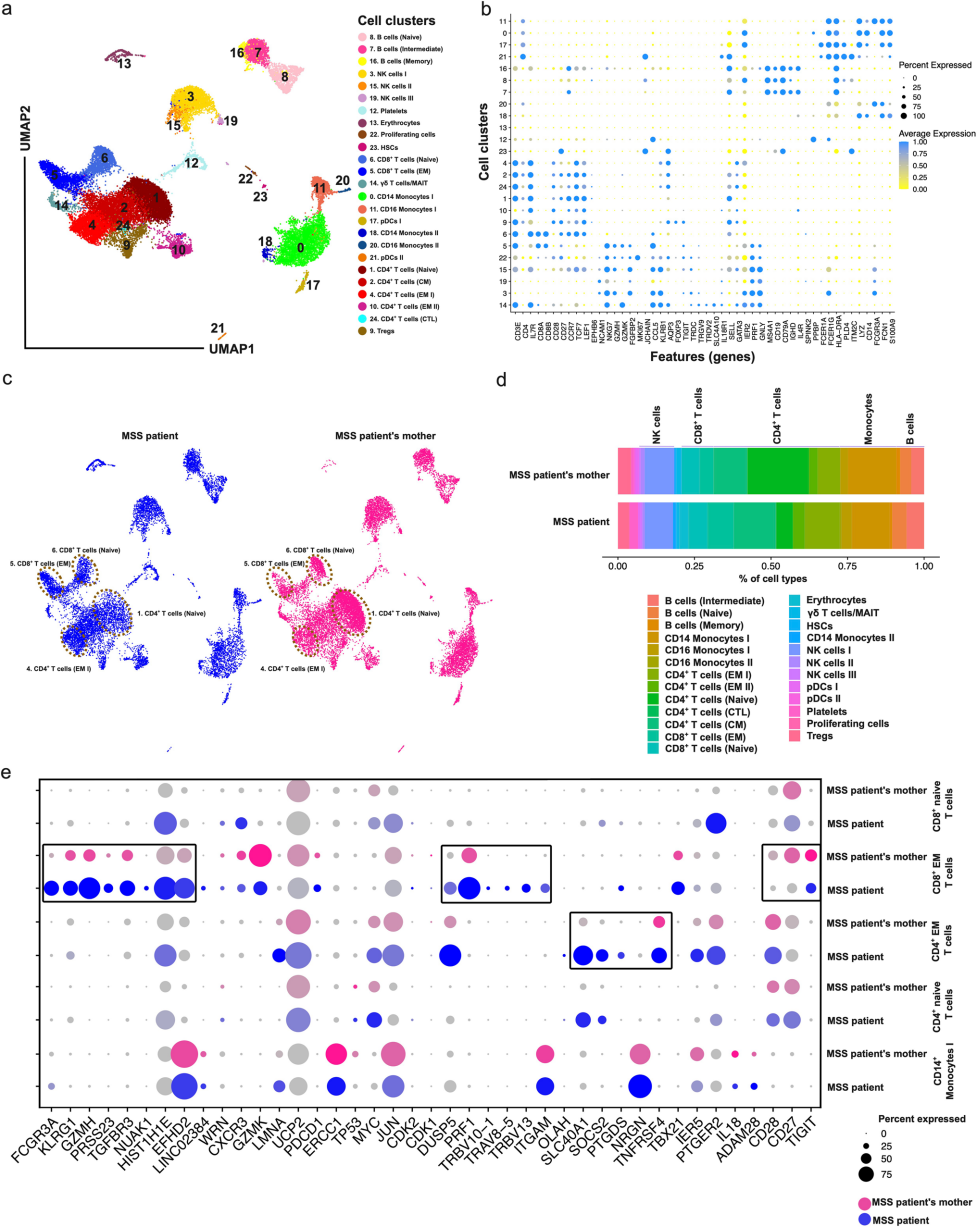
Impaired innate and adaptive immune system in the MSS patient. **a** Identification of individual cell clusters using single cell RNA transcript levels using Louvain clustering and data presented in UMAP plot for all the samples. **b** Detection of cell clusters based on important gene signature for individual cell types as shown on dot plot. X-axis represents individual cell clusters and Y-axis represents important feature genes for individual cell types based on the previous literature. **c** Independent UMAP plots for the MSS patient and her mother. Highlighted ovals show the visual change in number of cells based on RNA transcriptomics for the MSS patient and patient’s mother specially reduced naive CD4^+^ T cell (Naïve) and CD8^+^ T cells (Naïve) cells whilst increased CD4^+^ T cells (EM) and CD8^+^ T cells (EM) in lymphocyte compartment in the MSS patient. **d** Bar plot shows the percentage changes in different subsets of lymphocytes and monocytes in the MSS patient and mother of the patient. Reduced lymphocytes and monocytes mostly appeared in the MSS patient compared with her mother. **e** Selective ageing gene expression in the MSS patient. The dot plot shows the higher expression of several genes such as *LMNA, PDCD1, GZMK, HIST1H1E,* and *CXCR3* with ageing phenotypes in MSS patient compared with her mother. Selective TCR clonal expansion (*TRBV10-1, TRBV13*, and *TRAV8-5*) in CD8^+^ EM T cells from MSS patient in CD8^+^ T (EM) cells. Selective gene expression in inflammatory (*SOCS2, OLAH,* *ZNF642*), activation (*SLC40 A1, IER5*), prostaglandins (*PTGDS, PTGER2*), and histones (*HIST1H1E*) in CD4^+^ T (EM) cells

MULTI-omics approaches are being applied to solve likely rare genetic diseases to direct clinical management (Delgado-Vega 2024; Baysoy et al. 2023; Lunke et al. 2023), however, only a few cases have been described applying sc-RNA-seq analysis (Hua et al. 2023; Kim et al. 2020) but not sc-MULTI-omics-CITE-seq. Thus, sc-MULTI-omics-CITE-seq coupled with antigen-specific receptors (gene expression, surface protein immune cell markers, TCR, and BCR) analysis may be a unique and novel powerful approach to study nano-rare diseases. Here, we applied this method using single-cell suspensions of PBMCs in a nano-rare disease with no access to another patient. After the QC (Suppl. Figure 1a-b), unsupervised clustering and *uniform manifold approximation and projection* (*UMAP*) plot analyses were performed (Fig. 1a). Cluster identities were determined based on the expression of the established canonical markers (Fig. 1b and Suppl. Figure 1c-d), which revealed the successful capture of major blood cell subsets (Fig. 1a-b). In comparing the MSS patient with the patient’s mother (control), we identified abundance of immune cells (Fig. 1c) that reflected a bias towards the CD14^+^ monocytes I, plasmacytoid dendric cells II and CD4^+^ naïve T cells (all the three cell types lower in the MSS patient) as well as effector memory either CD4^+^ or CD8^+^ (EM; higher in the MSS patient) T cell subsets (Fig. 1d, and Suppl. Figure 1e-f), which is a sign of a progressive aging phenotype. Later, we projected our data with a well-annotated proteogenomic reference multimodal single cell database (Hao, et al. 2021) (Suppl. Figure 2a-c) and identified that the percentage of the same cell clusters was reduced in the MSS patient, akin to unsupervised clustering (Suppl. Figures 1d and 2d).

To understand the biological significance of transcriptional changes, we first performed differentially expressed genes analyses (DEGs) (Suppl. Figure 3a-c), focusing on CD14^+^ monocytes I, and identified that several genes were differentially regulated (upregulated and downregulated) in the MSS patient compared with control. Metascape analysis (Zhou et al. 2019) revealed that several prominent pathways were enriched in CD14^+^ monocytes type I (based on upregulated genes; adjusted *p* value ≤ 0.05), including hemostasis, clotting cascade, platelet activation, cytokine signalling, and transcriptional dysregulation in cancer (Suppl. Figure 3a). Further, DEGs analysis of CD4^+^ EM or CD8^+^ EM T cells resulted in segregation of the MSS patient from her mother as in both the cell subtypes, we observed more gene expression in the MSS patient (Suppl. Figure 3b, c). Pathway analysis of CD8^+^ EM T cells revealed that MSS patient had a higher inflammatory response and cell-killing potency (Suppl. Figure 3b). Furthermore, several other pathways were also upregulated, including the circadian rhythm, regulation of Insulin-like Growth Factor (IGF) transport, antigen processing and presentation, and natural killer cell cell-mediated cytotoxicity (Suppl. Figure 3b). In contrast, CD4^+^ EM T cells showed several upregulated pathways related to integrin-mediated signalling, response to hormones, fatty acid metabolism, and response to toxic substances (Suppl. Figure 3c). Additionally, Gene Set Enrichment Analysis (GSEA) for gene ontology (GO) pathways and Kyoto Encyclopedia of Genes and Genomes (KEGG) pathways analyses were performed for DEGs (log_2_FC ≥ 0.2 and adjusted *p* value ≤ 0.05) in CD8^+^ EM T cells. Our data revealed that natural killer cell-mediated cytotoxicity, the ERBB2-ERBB4 signalling pathway, and transcriptional dysregulation in cancer pathways were upregulated in MSS patient (Supp. Figure 4).

Aging is characterized by chronic systemic inflammation accompanied by cellular senescence, immunosenescence, organ dysfunction, and age-related diseases (Li et al. 2023). Immune aging is defined based on the low abundance of either naïve CD4^+^ or CD8^+^ T cells and increased CD8^+^ or CD4^+^ EM T cells (Li et al. 2023; Mogilenko et al. 2022; Lee et al. 2022). In the MSS patient, we found several markers (*FCGR3 A, KLRG1, GZMH, PRSS23, TGFBR3, NUAK1, LINC02384, PDCD1*, *HIST1H1E,* and *DUSP5*) upregulated in CD8^+^ and CD4^+^ EM T cells (Fig. 1e). Many genes, such as *CCL2* and *UCP2,* were upregulated in progeria (Caliskan et al. 2022) whilst *CD27, CD28*, and *TIGIT* were dysregulated in senescent cells (Martyshkina et al. 2023). We found that *LMNA, UCP2, CD28, GZMK*, and *PDCD1* were upregulated in CD8^+^ EM T cells (Fig. 1e). Furthermore, there was also clonal expansion of certain TCR-α and -β chains (*TRBV10-1, TRAV8-5,* and *TRBV13*), which also suggested that MSS patient has aged immune cell types and/or certain inflammatory pathologies (Fig. 1e). Furthermore, we identified that several genes related to iron export, keratin, inflammation, prostaglandins, and histone modification (*SLC40A1, SOCS2, PTGDS, NRGN, TNFRSF4, PTGER2, IER5*, and *HIST1H1E*) that were also upregulated in CD4^+^ EM T cells in the MSS patient (Fig. 1e).

**Fig. 2:**
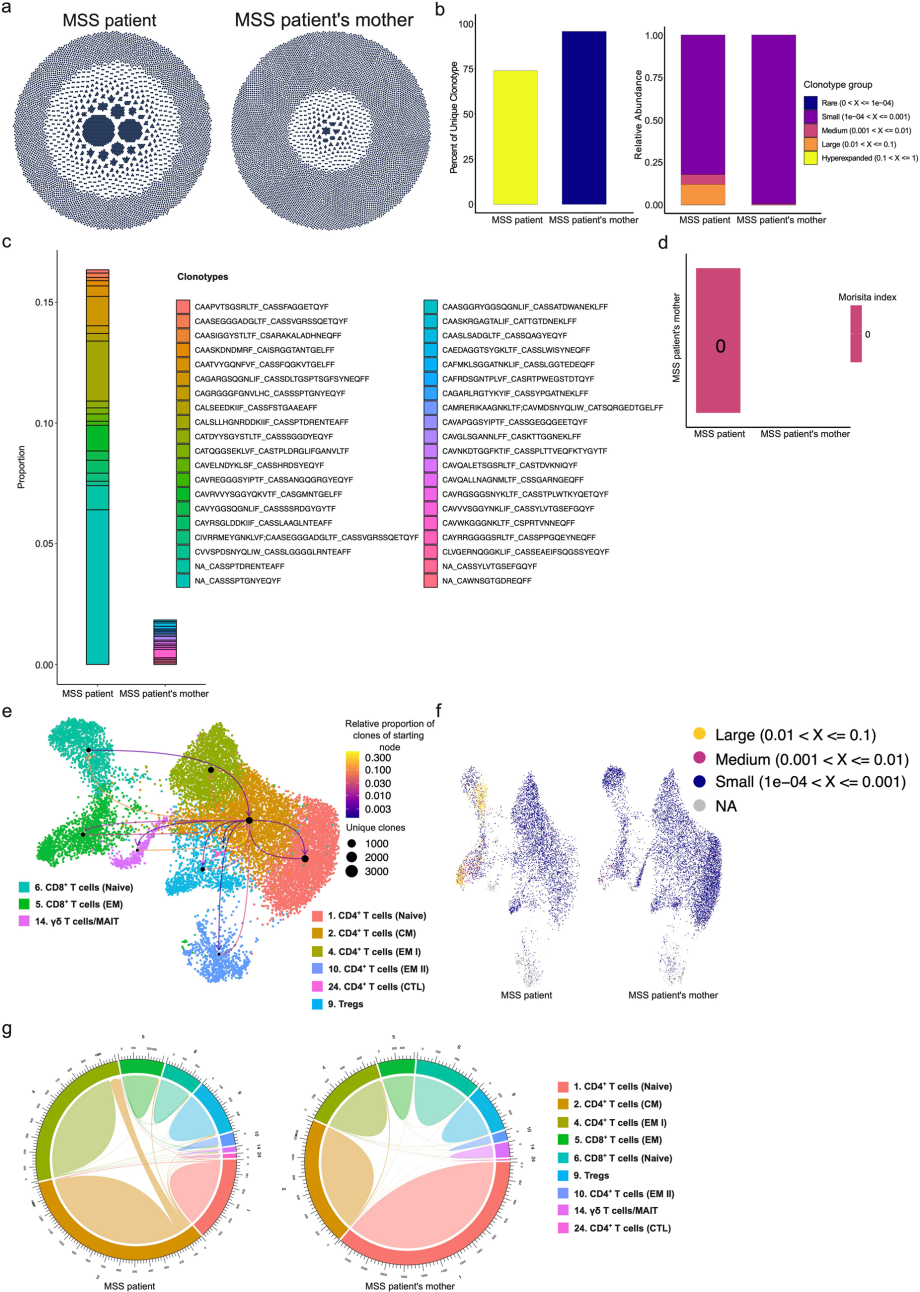
CD8^+^ EM TCR clonal expansion in MSS patient. **a** Circle dot plots denote TCR abundance, bigger dots represent the clonal expansion. The MSS patient had higher TCR clonal expansion compared to her mother. **b** Percentage of unique clonotypes and relative abundance of clonotypes in MSS patients compared with her mother. The MSS patient has restricted clonotypes compared to her mother. **c** Proportion of highly abundant (top 20) clonotypes in the MSS patient and her mother. **d** Similarity index between MSS patient and control (her mother). No similar TCR clonotypes were found between MSS patient and her mother. **e** Communications of TCRs with other TCR clonotype and cell clusters (**f**) Representation of clonal expansion on UMAP plot in the MSS patient and her mother. Large clonotypes (> 0.1%) were only found in the MSS patient. **g** Tracking of TCRs in different clusters in the MSS patient and her mother

TCRs are generated by somatic recombination which contain a highly unique repertoire (89%—92%) in each individual, however, individuals do tend to share 8% of TCRβ—or 11% of TCRα-chain clonotypes (Soto et al. 2020; Jiang et al. 2018). Furthermore, healthy monozygotic twins are considerably identical in their TCR repertoire than unrelated individuals (Rosati et al. 2020). Previously, it was demonstrated that increasing age is associated with (a) reduced αβ TCR repertoire richness in CD8^+^ naïve T cells; (b) increased clonal expansion of CD8^+^ memory T cells, (c) increased overlap in TCR sequences in longitudinal samples memory CD8^+^ T cells, and (d) reduced distinction of TCR sequences between naive and memory CD4^+^ and CD8^+^ T cells as well as between CD4^+^ and CD8^+^ T cells (Sun 2022). To decipher if change in TCRs or BCRs repertoire richness could help us to predict progeria phenotype in MSS patients as well as whether TCRs or BCRs clonotype parallelism could disclose the resemblance with her mother due to close genetic relationship, we performed sc-TCR-seq and sc-BCR-seq coupled with gene expression analysis. Our data revealed that the MSS patient indeed had an increased expansion of paired αβ TCRs compared with control (Fig. 2a). Thus, the MSS patient appeared to have less unique clonotypes with mostly large- and medium-sized expansions (Fig. 2b). Furthermore, the MSS patient had no common sequence-expanded clonotypes to control (her mother), as reflected by the Morisita-Horn similarity index (Fig. 2c, d). Thus, it reflects that divergence in the repertoire selection. The top expanded clonotypes are shown in the paired form in CD8^+^ T cells (Fig. 2c-e; right and Suppl. Figure 5). Additionally, we also investigated how each clonotype interacted with other cell clusters. It appeared that the CD4^+^ CM T cell cluster clonotypes interacted with other cell types (Fig. 2e), and the clonal frequency of TCRs was higher in MSS patient, as shown in the UMAP plots (Fig. 2f). Finally, we demonstrated that CD4^+^ CM T cells communicate with all other cell types in patient with MSS (Fig. 2g).

Similarly, akin to TCR repertoire analysis, we also did not observe an increased expansion of BCR heavy (IGHV) or light (IGKV) chains in the MSS patient compared to the control, and similar levels of unique clonotypes were present (Suppl. Figure 6a). Surprisingly, some heavy chains were absent (*IGHV1-69–2*), whereas others were over-represented (*IGHV4-4, IGHV3-23,* and *IGHV1-3*) in the MSS patient (Suppl. Figure 6b). Some of the unique clonotypes were common among the MSS patient and control, but not the expanded ones, based on the Morisita similarity index and clonotype frequency expansion (Suppl. Figure 6c, d). Furthermore, only medium clonotypes were found to be available in patients’ mother, while small clonotypes were dominant in the MSS patient (Suppl. Figure 6e, f). Communication between different clusters was limited in the MSS patient compared with control (her mother) (Suppl. Figure 6e, g). Overall, BCRs showed limited changes in the patient with MSS and control.

## Discussion

Although highly specific as a recognizable syndrome, the (genetic) cause(s) of MSS have not yet been identified. This is largely due to its nano-rare occurrence with only 12 patients reported worldwide. Unfortunately, due to the early development of cancer, most of the patients died already. Here, we advanced our sparse knowledge of MSS by performing sc-MULTI-omics-CITE-seq (gene expression, protein expression, TCR, and BCR repertoire analysis) on a single MSS patient. In this unique study, we identified that immune cells, especially CD4^+^ T and CD8^+^ T cells, have a progeria-like pattern (limited naïve T cells and expanded TCR clonotypes) (Sun 2022; Young et al. 2023), which recapitulates the previously described clinical phenotype of MSS patients (NORD 2024; Passarelli, et al. 2018). However, this does not explain the clinical manifestations observed in patients with MSS.

At the transcriptomic level, we identified that in addition to genes already described in aging phenotypes in EM T cells such as *PDCD1*, *GZMH* and *CXCR3*, new genes (*NUAK1*, *LINC02384*, *CCL2*, *UCP2*, *SLC40 A1*, *SOCS2*, *PTGDS*, *NRGN*, *TNFRSF4*, *PTGER2*, *IER5*, and *HIST1H1E*) that could help us to predict the phenotype of the MSS patient. If a drug is administered to modulate these genes or pathways (CXCR chemokine receptor binding, complement, coagulation cascades, and transcription dysregulation in cancer) in immune T cells, the treatment response can easily be monitored based on the reversal of transcriptional hallmark signatures with specific immunomodulation for precision medicine approaches in nano-rare diseases. Histone proteins play an important structural and regulatory role in genome compaction, thus it is not surprising that histone functional dysregulation or aberrant levels of histones could lead to severe consequences for multiple cellular processes and might ultimately govern development or contribute to cell transformation (Flex et al. 2019). Furthermore, *HIST1H1E*, encodes the Histone H1 protein, has been implicated in accelerated cellular senescence, premature aging and cognitive decline (Flex et al. 2019). We identified that *HIST1H1E* was highly upregulated in CD8^+^ EM T cells, and it could be attributed with progressive aging in the MSS patient.

The inferred disease-enriched pathways recapitulated known biology and highlighted notable cell-disease relationships in monocytes and lymphocytes (CD4^+^ and CD8^+^ T cells). For instance, in monocytes type I, gene signatures identified that patients with MSS might have increased hemostasis, activated platelets, increased neutrophil extracellular trap formation, cell migration, muscle physiology, and cancer-associated pathways, which could be dysregulated and can predict the next possible disease outcome. Keeping this in mind, our patient developed HCC six months after blood sampling; therefore, longitudinal sample analysis could be advisable if the patient is undergoing treatment for HCC. Gene pathway analysis of CD8^+^ EM T cells suggested that these cells are highly pro-inflammatory in nature with expanded TCRs, which could damage the body and fasten the aging process of the patient. In addition to inflammation, it appears that CD8^+^ EM T cells could also have a role in the modulation of insulin signalling by changing antigen presentation, which could be reflected by ongoing T3DM in MSS patients. In contrast, CD4^+^ EM T cells could be involved in sensing the changes in fatty acid or estradiol metabolism and integrin-mediated signalling pathways in the MSS patient. Estradiol plays an indispensable role in metabolism regulation, including glucose homeostasis, lipid metabolism, and energy balance (Camon et al. 2024). Further, declining estradiol can contribute to age-related metabolic dysfunctions like insulin resistance and obesity. Our data suggest that intact levels of estradiol gene pathways in MSS patient. Thus, measurement of estradiol levels in MSS patients could help us to understand the progenic phenotype, thus, further studies are warranted.

Protein expression, TCR clonotype expansion, and changes in antibody production due to altered BCRs could provide valuable information. Our TCR analysis identified that certain inflammatory clones were expanded in the MSS patient; however, the establishment of TCR expansion with aging has not been determined due to limited single-cell TCR studies in the literature. Based on the BCR sub-study, we did not identify any expanded BCRs; however, we noticed that certain *IGHV* chains were missing in the MSS patient, but the *IGHV3-23* clone was expanded. However, further studies are propositioned to understand these datasets.

## Conclusions

Overall, our sc-MULTIomics-RNA-seq analysis deciphered the unknown role of immune cells in the aging process and could be the first warning sign of a developing cancer phenotype. Studies from other patients with MSS will certainly help to understand the pathomechanism of MSS patient in more detail. We pitch this approach may become a general strategy to analyse nano-rare patients’ diagnosis or personalized medicine.

## Limitations

MSS is a nano-rare disease; therefore, its comparison with other patients is certainly advisable. The results may be patient-specific rather than MSS-specific, which could constrain the generalization of the conclusions reported in this report. However, we believe that our study will create a scientific interest in the medical community and raise public awareness to participate in the study to understand the pathomechanism of this and other nano-rare syndromes and highlight the great potential of sc-MULTI-omics-CITE-seq coupled with TCR/BCR analysis as a valuable tool for genomic-based precision medicine in single patients.

## Supplementary Information

Below is the link to the electronic supplementary material.

Supplementary file1 (DOCX 2.31 MB)

## Data Availability

• The data generated by scRNA-sequencing from this study is available to download through the public repository *via* the following accession number or link: DOI: 10.5281/zenodo.10894666. • This study did not report any original code. All detailed analysis pipelines are available at GitHub repositories (https://github.com/ysinghbt/MSS). • Any additional information required to re-analyse the data reported in this paper is available from the lead contact upon reasonable request.

## Abbreviations

MSS: Mulvihill-Smith syndrome
sc-MULTIomics RNA-seq: single cell (sc)-MULTI-omics-RNA-sequencing
UMAP: Uniform manifold approximation and projection
DEGs: Differentially expressed genes
GSEA: Gene Set Enrichment Analysis
KEGG: Kyoto Encyclopedia of Genes and Genomes
