# Supplementary material for: sc-MULTI-omics approach in nano-rare diseases: understanding the pathophysiological mechanism of Mulvihill-Smith Syndrome
Source: Funct Integr Genomics. 2025 May 9;25(1):101. doi: 10.1007/s10142-025-01608-y (PMC12064584; doi:10.1007/s10142-025-01608-y)
Supplement: Supplementary file 1 — Supplementary file1 (DOCX 2.31 MB) [file 10142_2025_1608_MOESM1_ESM.docx]

***Brief Report***

**sc-MULTI-omics approach in nano-rare diseases: Understanding the pathophysiological mechanism of Mulvihill-Smith Syndrome**

Angelika Riess^1^, Cristiana Roggia^1^, Antje Schulze Selting^1,2^, Vladislav Lysenkov^1,2^, Stephan Ossowski^1^, Nicolas Casadei^1,2^, Olaf Riess^1,2*^, Yogesh Singh^1,2,3*^

^1^Institute of Medical Genetics & Applied Genomics, University Hospital Tübingen, Tübingen University, Tübingen, Germany

^2^NGS Competence Centre Tübingen, University Hospital Tübingen, Tübingen University, Tübingen, Germany

^3^Research Institute of Women’s Hospital, University Hospital Tübingen, Tübingen University, Tübingen, Germany

*Correspondence to: Dr Yogesh Singh or Prof Olaf Riess, Institute of Medical Genetics & Applied Genomics, University Hospital Tübingen, Tübingen University, Tübingen, Germany or by Email: [olaf.riess@med.uni-tuebingen.de](mailto:olaf.riess@med.uni-tuebingen.de), [yogesh.singh@med.uni-tuebingen.de](mailto:yogesh.singh@med.uni-tuebingen.de)

**Supplementary information**

**Materials and Methods**

*Patient demographics*

Both, MSS patient and mother, signed the consent for the Genome+ study (ClinicalTrial.gov-Nr.: NCT04315727 and project Nr.: 635/2014BO1). MSS was diagnosed based on clinical findings of short stature, older appearance, multiple pigmented nevi, and microcephaly at the age of 1 year (2006) and she is currently 19 years old and not doing very well. Later, the patient developed monolateral keratoconus, Marcus-Gunn-Syndrome, hearing loss, vitamin D deficiency, mild hypercortisolism, and diabetes mellitus with very high insulin resistance (T3DM). Furthermore, she experienced a decline in cognitive ability and later underwent hemihepatectomy due to the transformation of already known liver adenomas into hepatocellular carcinoma (HCC). Recurred infections were ruled out. In this duo study, the patient with MSS and her mother were recruited for sc-MULTIomics-RNA-seq analysis on 06/2022. A few months later, the MSS patient was diagnosed with T3DM (10/2022), and 6 months later (12/2022) with HCC.

*Blood collection, PBMC isolation and sample preparation for single cell* ***C****ellular* ***I****ndexing of* ***T****ranscriptomics and* ***E****pitopes by Sequencing (CITE-seq)*

PBMCs were isolated using the standard Ficoll method^1^. We used fresh PBMCs (0.5x10^6^) for the single cell CITE-seq preparation. We resuspended PBMCs in cold PBS and filtered through 40 µm cell strainers (Falcon, USA) to remove potential clumps and large particles. Later, PBMCs were then incubated for 10 min with Fc receptor block (#422302; TruStain FcX, BioLegend, USA) to block nonspecific antibody binding. Subsequently, PBMCs were incubated with a mixture of 137 Feature barcode (FB) antibodies labelled with unique molecular barcodes, including seven isotype barcode antibodies (TotalSeq™-C Human Universal Cocktail, V1.0; #399905, BioLegend, USA) for 30 min at 4 °C, as recommended by the manufacturer. After incubation with antibodies for 30 minutes at 4 ^0^C, PBMCs were washed four times (centrifugation (~400xg 5 min at 4 °C and supernatant exchange) with cell staining buffer (#420201; BioLegend, USA), and cell viability was estimated using TN 184 DeNovix Acridine Orange/Propidium Iodide Assay kit (DeNovix, USA) and Celldrop (DeNovix, USA). PBMCs viability was greater than 90% in both MSS patient and control samples.

*Single cell CITE-seq, TCR and BCR sequencing (sc-MULTIomics-seq) library and sequencing*

We followed the 10x Genomics’ sample preparation protocol for 5’GEM V2 kits PN-1000425 (gene expression (GEX), feature barcode antibodies (FB), TCR receptor (TCR), and B cell receptors (BCR)) with slight modifications. Briefly, 30,000 FB-labelled PBMCs were loaded onto a 10x chromium controller (10x Genomics) together with RT enzymes and 5’ gel beads in emulsion (GEM) beads to make single cell suspensions together with GEM beads. Once the single cell separation process was completed, single cell RNA was converted into single cell cDNA. Single cell cDNA was purified and amplified for nine PCR cycles to obtain sufficient products for GEX, FB, TCR, and BCR library preparation. Individual libraries were prepared, and quality checks (QC) were performed as recommended by the manufacturer. scRNA-seq libraries were constructed using Chromium Single Cell 5′ v.2.0 Reagent (PN-1000263, Human T Cell, 1000252; Human B Cell, 1000253; 10X Genomics), and a unique sample index was included in each sequencing library. Finally, sequencing was performed using a paired-end 200 bp reading strategy on an Illumina platform. An appropriate amount was loaded onto the sequencing cell flow S2 or S4 (Illumina) to obtain >35,000 reads/cell for GEX, >10,000 reads/cell for FB, >10,000 reads/cell for TCR, and >10,000 reads/cell for BCR.

*Single cell data processing, alignment and generation of count matrices files*

Sequencing output files (FASTQ files) were processed using a 10x cell ranger pipeline to obtain count matrix files (filtered feature barcode matrices) for the downstream data analysis. Briefly, both samples were demultiplexed, barcode processed, 5' gene counted, V(D)J transcript sequence assembled and annotated, and Feature Barcode analysed. Cell Ranger (v.3.0.2) was used to align reads to reference (GRCh38-3.0.0) and generate feature-barcode matrices from the Chromium scRNA-seq output.

*Data analysis for sc-MULTIomics-RNA-seq* experiment

Gene expression and cell annotations

Filtered feature gene expression matrices generated per sample were analyzed with the Seurat 5.01 package in RStudio (4.4.2). The filtering steps for high-quality single cells included cells expressing >200 genes, cells with >800 detected molecules (the total number of unique molecular indices (UMIs) detected), and cells with a mitochondrial gene count percentage of <10%. Genes that appeared in less than three cells were excluded. These conditions help to identify cells which are well captured and are likely to provide reliable information about gene expression patterns in individual cells. This yielded a total of 25,157 good quality cells. QC analysis before and after filtration of cells was performed for total genes, ribosomes, and mitochondria (Suppl. Fig. 1).

Based on the clean scRNA-seq data after quality control (QC) checks, gene expression profiles were normalized for each cell using the Log-Normalization method with the Seurat NormalizeData function. The FindVariableFeatures function was applied to identify highly variable genes using the default parameters. Next, we scaled the data using the ScaleData function and performed principal component analysis on the scaled data using the RunPCA function with default parameters. A canonical correlation analysis (CCA) integration algorithm was used for batch correction^2,3^. We constructed a shared nearest neighbor graph using the FindNeighbors function and clustered cells using the Louvain algorithm with the FindClusters function, with the resolution set to 0.8, based on CCA integration reduction^4^. Finally, the RunUMAP function facilitates the visualization of all cells.

*Cell-type annotation and cluster marker identification*

The FindAllMarkers function was used to identify the marker genes for each cluster. Clusters were then classified and annotated based on the marker gene expression of each cell subset. In brief, clustering (resolution, 0.8) identified 25 major and minor cell subtypes, including CD4^+^ T cells, CD8^+^ T cells, γδ T cells/MAIT, NK cells, B cells, CD14^+^ monocytes, CD16^+^ monocytes, monocyte-derived dendritic cells, plasmacytoid dendritic cells, and megakaryocytes/platelets (Suppl. Fig. 1c, d). Single cells expressing two sets of well-studied canonical markers of major cell types were labelled as doublets and excluded from subsequent analyses.

*Multimodal reference mapping*

In the process of mapping query datasets to annotated references in Seurat, we mapped our PBMC dataset obtained from the MSS patient and control to the CITE-seq reference of 162,000 PBMC measured using 228 antibodies^2^. We demonstrate how supervised analysis guided by a reference dataset can help enumerate cell states that can be challenging to identify using unsupervised analysis. We loaded the published reference data set^2^ and visualized the pre-computed UMAP (more information is available in Suppl. Fig. 2). The reference was normalized using the SCTransform function, and the same approach was utilized to normalize with the query data. We then found anchors between the multimodal reference dataset with our query dataset. Using a precomputed supervised PCA (spca) transformation, we took advantage of supervised PCA for CITE-seq datasets, which led to the transfer of cell type labels and protein data from the reference to our dataset. We projected our query data onto the UMAP structure of the reference dataset using annotated cells (Suppl. Fig. 2).

*Identification of differential expressed genes across the MSS patient and control*

To perform comparative analyses across the MSS patient and control group, we used the bulk of Seurat’s differential expression features which was accessed through the [FindMarkers()](https://satijalab.org/seurat/reference/findmarkers) function. By default, Seurat performs differential expression (DE) testing based on the non-parametric Wilcoxon rank-sum test. To test for DE genes between the two specific groups of cells (MSS patient and control), we specified the ident.1 and ident.2 parameters. Differentially expressed genes (DEGs) from each cell subtype were filtered based on the following criteria: absolute fold change ≥0.2 and adjusted p value ≤0.05. We evaluated the DEG profiles of the three cell types (CD14 monocyte I, CD8^+^ EM T, and CD4^+^ EM T cells), and compared their gene expression patterns. We highlighted genes that exhibited dramatic differences between MSS patient and control (Suppl. Fig. 3; left volcano plots) based on log_2_FC and, adjusted p value correction utilized the Bonferroni correction.

*Metascape data analysis*

The Metascape web browser tool utilizes a well-adopted hypergeometric test and Benjamini-Hochberg p-value correction algorithm to identify all ontology terms which encompass a statistically greater number of genes in common with an input list than expected by chance^5^. Thus, we used the input of highly upregulated genes (top 100 genes from the DEGs list) from monocytes I, CD8^+^ EM, and CD4^+^ EM T cells that were derived from the MSS patients compared with the control based on log_2_FC and adjusted p value correction utilized Bonferroni correction using all features in the dataset. These genes were subjected to Metascape web browser analysis^5^ to identification of different biological pathways (Suppl. Fig. 3; right bar plots).

Gene set enrichment analysis (GSEA) for Gene Ontology (GO) and Kyoto Encylopedia of Genes and Genomes (KEGG) pathways

GSEA was used to identify whether a pre-defined set of genes (log_2_FC≥0.2 and p value ≤0.05; based on false discovery rate (FDR) correction) in a particular subset of cells showed statistically significant, concordant differences between the MSS patient and control. We used “Clusterprofiler v3.20”, pathwiew, enrichplot, wordcloud, msigdbr R packages for data analysis. Gene set enrichment analysis was performed using the gseGO function with default values. “DOSE” R-package was used for making dotplots for gse or KEGG data for top 10 pathways followed by GSEA plots for the selected genesetIDs (Suppl. Fig. 4). Significantly activated or inhibited KEGG pathways were selected with absolute NES≥1 and adjusted p value ≤0.05, as the threshold. For each cell subset, Gene ontology (GO) enrichment analysis was performed on the previously selected DEGs. The significantly changed GO entries were filtered with adjusted p value≤0.05 and count ≥2 as threshold.

*TCR and BCR repertoire sequencing and analyses*

The scTCR and scBCR repertoire libraries for both the samples (10× Genomics) were prepared and profiled with paired-end sequencing (2 × 150 bp) on the Illumina Nova Seq 6000 platform. The Cell Ranger V(D)J pipeline (10× Genomics, v.3.0.2) was used to mark the TCR and BCR gene expression and clonotypes identification. The TCR and BCR repertoire sequencing data were filtered with the following criteria: productive is “True”; high_confidence is “True”; umis ≥1; and raw_consensus_id is not “None”. Only T cells with at least one TCR α-chain and one TCR β-chain were used to identify the TCR clonotypes for each T cell. If two or more TCR α-chains or β-chains were assembled in a specified T cell, the highest-expressed (UMI or reads) TCR α-chain or β-chain was defined as the dominant TCR α-chain or β-chain. A clonotype was defined by a unique TCR α(s)-TCR β(s) pair (including CDR3 nucleotide sequences and rearranged VJ genes) within a T cell. BCR clonotypes were identified in a manner similar to TCR clonotypes. In brief, only B cells with at least one productive heavy chain (IGH) and one productive light chain (IGL or IGK) were retained for further analysis. Each unique IGH and IGL/IGK pair within a B cell was defined as a BCR clonotype. If there were two or more IGH and IGL/IGK assembled in each B cell, the highest expressed IGH and IGL/IGK chains were regarded as the dominant IGH and IGL/IGK chain^6^. Most of the downstream analysis for TCR and BCR repertoire was performed using scRepetoire pipeline^7^ and described earlier by others^8^.

*Statistical analyses*

Statistical analyses were performed using R software (v.4.3.0). This is a single patient-specific report duo study report. Therefore, major statistical analysis was not performed. However, significant differences in gene expression were determined by non-parametric Wilcoxon rank sum test and the P values were adjusted with FDR utilizing Bonferroni correction.

*Data availability*

All the detailed analysis pipeline is available on github repositories (<https://github.com/ysinghbt/MSS>) and data is available on Zenodo (**DOI: 10.5281/zenodo.10894666**). Further information on data availability can be provided upon reasonable request from the corresponding authors.

**Supplementary Results**

**
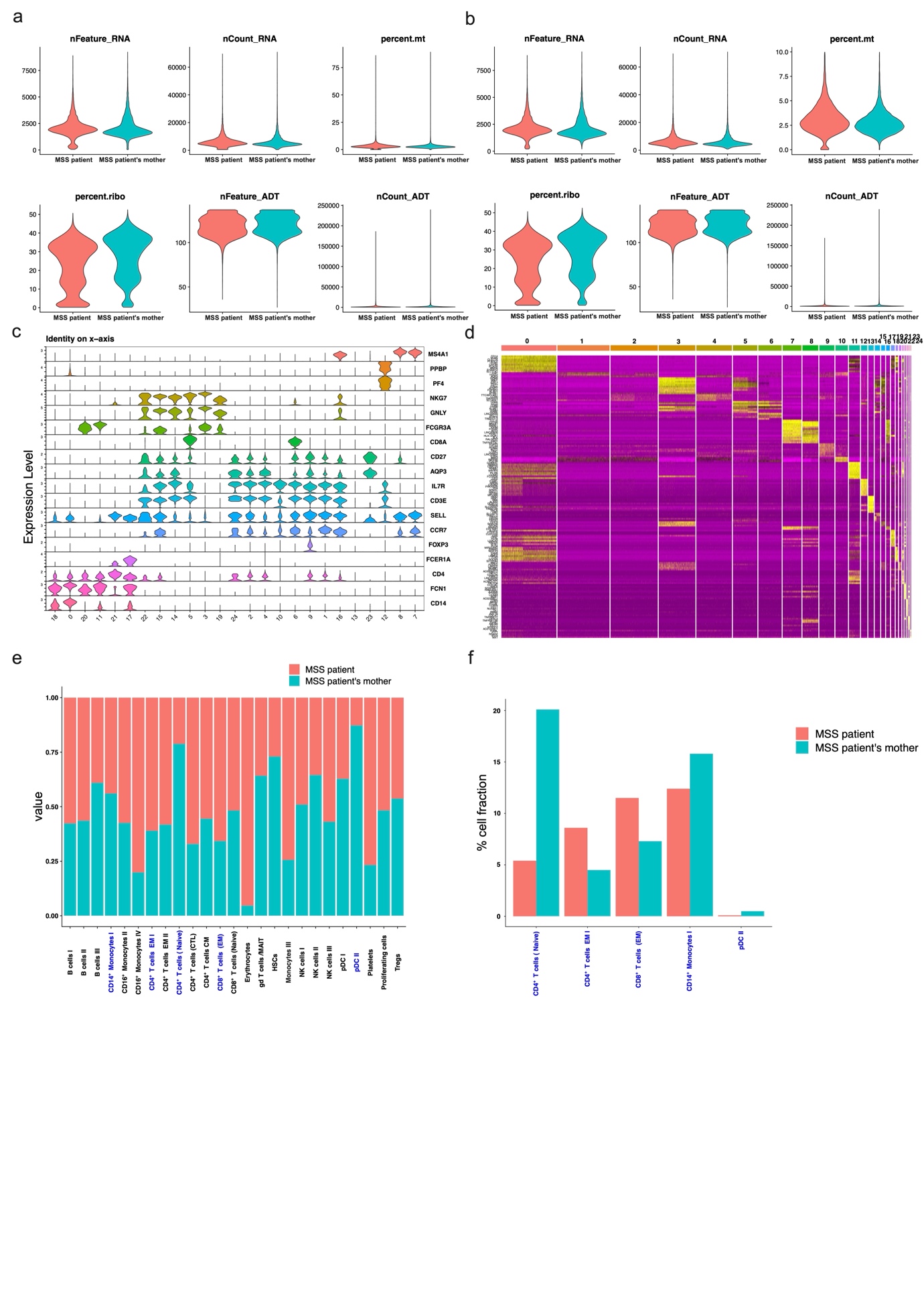
**

**Suppl. Fig. 1** Quality control of sc-RNA-transcriptomics, cluster identification and percentage of clusters in the MSS patient and patient’s mother. (a, b) Number of features, nCount, percentage mitochondria, percentage of ribosomal RNA, nFeature and nCount of feature bar antibodies pre- and post-processing of the data after basic quality check. (c) Key marker genes used for identification of cell clusters in PBMCs. (d) Top 10 genes used for cluster classifications shown in heatmap. (e) Percentage of each cluster between the MSS patient and control. (f) Highly dysregulated percentage of cells in the MSS patient including CD4^+^ naïve T cells, CD4^+^ EM I T cells, CD8^+^ EM T cells, CD14^+^ Monocytes I and pDC II.


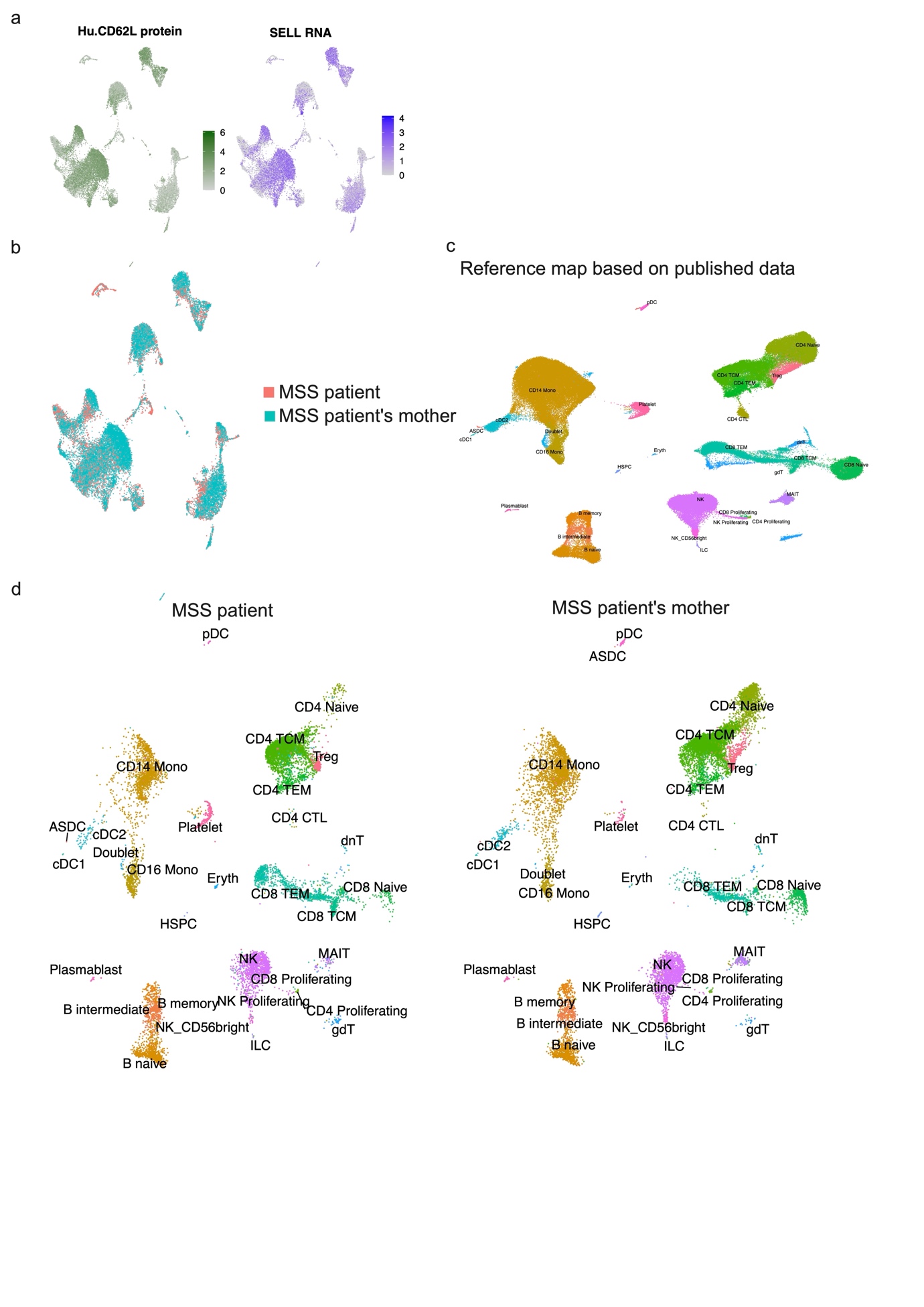


**Suppl. Fig. 2** Cluster confirmation with antibody expression (proteogenomic), alignment of expression and clusters in the MSS patient. (a) Cluster confirmation with protein expression for naïve marker based on CITE-seq (proteogenomic). (b) Alignment of the MSS patient and control in UMAP – overlay of cell clusters. (c) Reference database based on 228 protein expression for cell clusters derived from previously published data set of 228,000 cells^2^. (d) Percentage of each cluster between the MSS patient and her mother based on reference database as described in c.

**
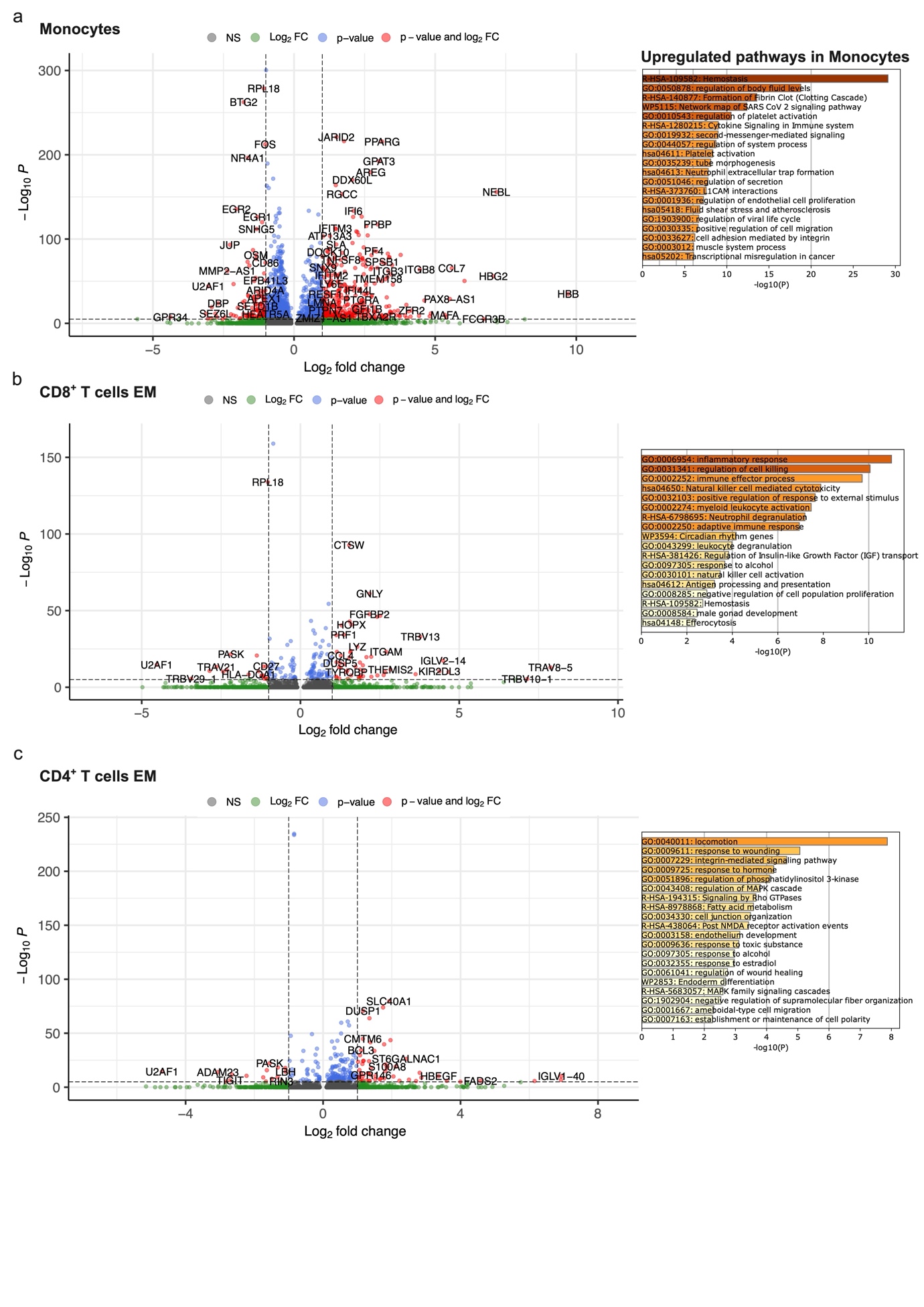
**

**Suppl. Fig. 3** Deregulated gene expression in innate and adaptive immune system in the MSS patient. (a) Volcano plots (left plot) for differential gene expression of the MSS patient compared with her mother in CD14^+^ monocytes I. Metascape pathway analysis (right bar plot) highlights the major biological pathways changes related to hemostasis, formation of firbin clot, regulation of platelet activation and cytokine signalling etc. based on upregulated genes found in MSS patient. (b) Similar analysis was performed for CD8^+^ T cells (EM) as well as for CD4^+^ T cells (EM). Volcano plots for (left plot) differential gene expression in the MSS patient and her mother for CD8^+^ T CM cells. Metascape pathway analysis (right bar plot) highlights the pathways involved in inflammation were activated (inflammatory response and regulation of cell killing) based on upregulated genes. (c) Volcano plots (left plot) show the differential gene expression in the MSS patient and control for CD4^+^ T CM cells. Metascape pathway analysis (right bar plot) highlights the activation of integrin-mediated signalling pathway.

**Suppl. Fig. 4** GSEA and KEGG pathways in CD8^+^ EM T cells in the MSS patients. (a) Gene set enrichment pathways which are activated and suppressed in the MSS patients. (b) KEGG pathways which are activated and suppressed in the MSS patients.


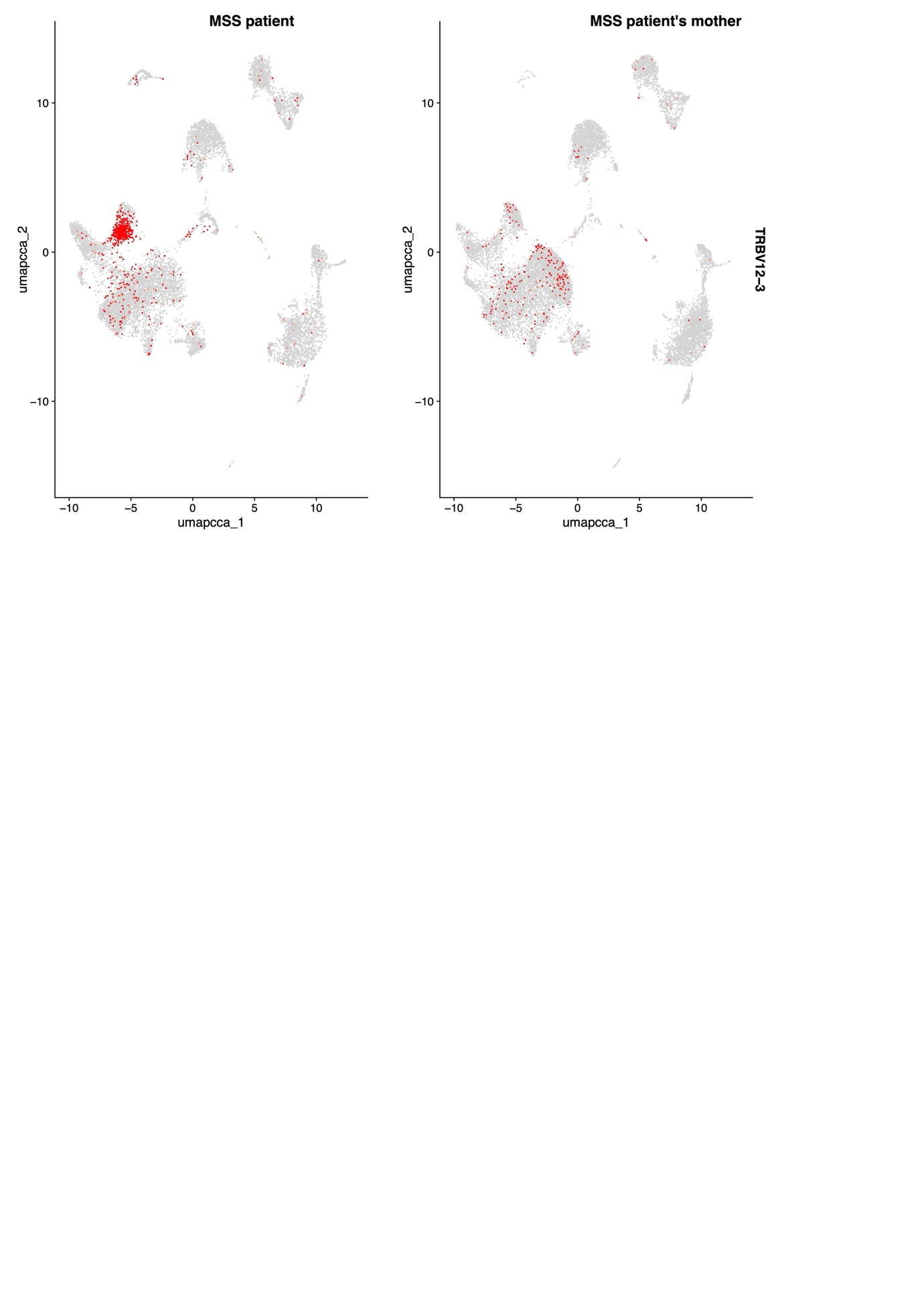


**Suppl. Fig. 5** TCR clonal expansion of TRBV12-3 clonotype in the MSS patient.


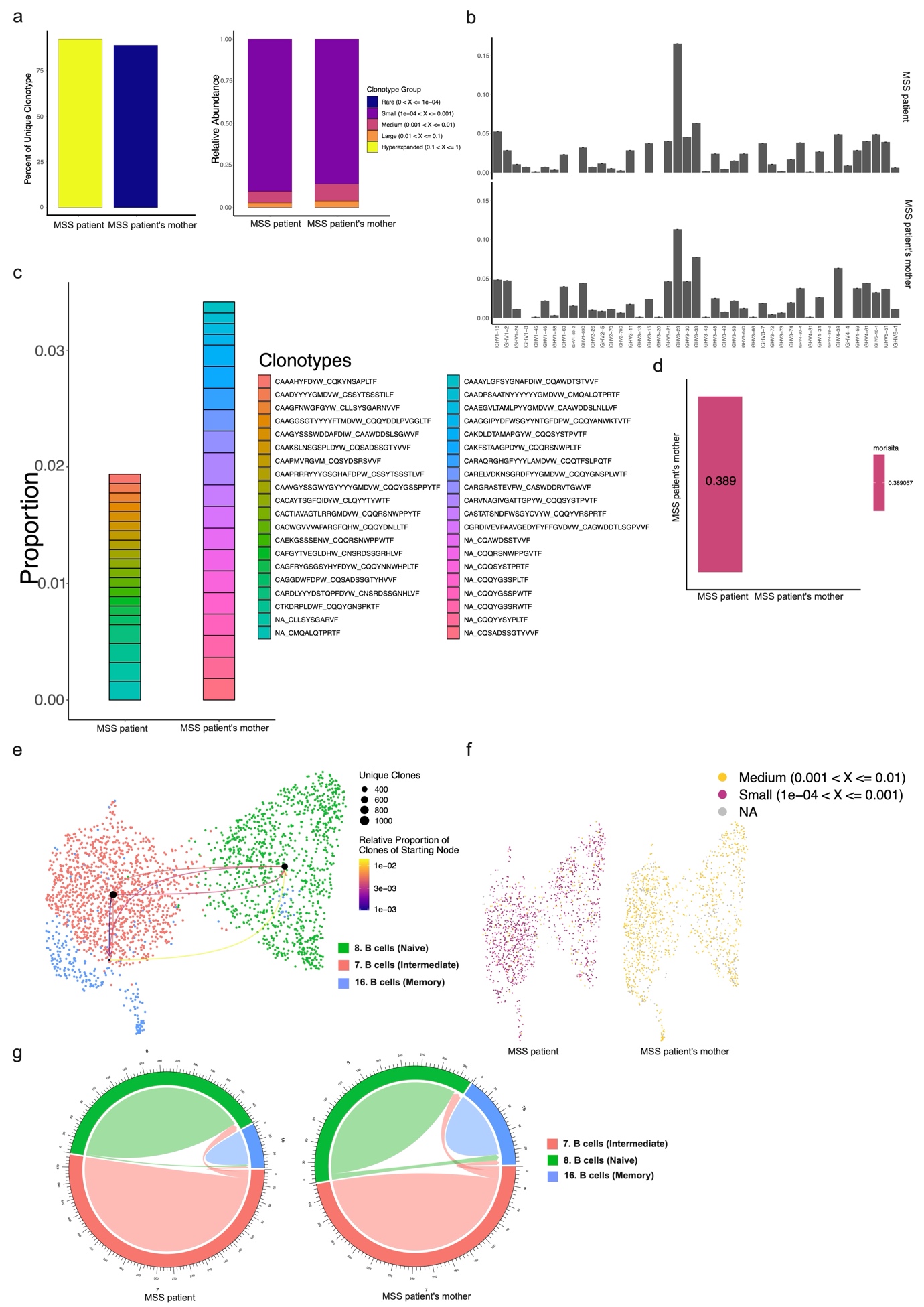


**Suppl. Fig. 6** BCR clonal expansion in the MSS patient. (a) Percentage of unique BCR clonotypes and relative abundance in the MSS patients compared with her mother. No apparent difference was observed in unique BCR clonotypes or their relative abundance. (b) Abundance of heavy chain of BCRs (c) Proportion of top 20 BCR clonotypes in the MSS patient and her mother. (d) Some common BCR clonotypes were present based on Morisita-Horn similarity index (38% percentage similarity) between MSS patient and her mother. (e) BCR communication with different B cell clusters. (f) Representation of clonal expansion on UMAP plot in B cell compartment in the MSS patient and her mother. The MSS patient had mostly small and medium clonotypes whilst her mother had mostly medium clonotypes abundance. (g) Tracking of BCRs in different clusters.

**References**

1. Singh, Y.*, et al.* SARS-CoV-2 infection paralyzes cytotoxic and metabolic functions of the immune cells. *Heliyon* **7**, e07147 (2021).

2. Hao, Y.*, et al.* Integrated analysis of multimodal single-cell data. *Cell* **184**, 3573-3587 e3529 (2021).

3. Stuart, T.*, et al.* Comprehensive Integration of Single-Cell Data. *Cell* **177**, 1888-1902 e1821 (2019).

4. Arevalo, J.*, et al.* Evaluating batch correction methods for image-based cell profiling. *Nat Commun* **15**, 6516 (2024).

5. Zhou, Y.*, et al.* Metascape provides a biologist-oriented resource for the analysis of systems-level datasets. *Nat Commun* **10**, 1523 (2019).

6. Wang, Y.*, et al.* Integrating single-cell RNA and T cell/B cell receptor sequencing with mass cytometry reveals dynamic trajectories of human peripheral immune cells from birth to old age. *Nat Immunol* **26**, 308-322 (2025).

7. Borcherding, N., Bormann, N.L. & Kraus, G. scRepertoire: An R-based toolkit for single-cell immune receptor analysis. *F1000Res* **9**, 47 (2020).

8. Andreatta, M., Gueguen, P., Borcherding, N. & Carmona, S.J. T Cell Clonal Analysis Using Single-cell RNA Sequencing and Reference Maps. *Bio Protoc* **13**, e4735 (2023).
